# Mitochondrial genotyping of an endangered bitterling *Acheilognathus typus* (Cyprinidae)

**DOI:** 10.3897/zookeys.623.8981

**Published:** 2016-10-11

**Authors:** Kenji Saitoh, Kentaro Shindo, Yasufumi Fujimoto, Kiyotaka Takahashi, Tetsuo Shimada

**Affiliations:** 1 National Research Institute of Fisheries Science, Japan Fisheries Research and Education Agency, Fukuura 2-12-4, Kanazawa, Yokohama 236-8648, Japan; 2 The Miyagi Prefectural Izunuma-Uchinuma Environmental Foundation, Shikimi 17-2, Kamihataoka, Wakayanagi, Kurihara, Miyagi 989-5504, Japan; 3 Society for Shinaimotsugo Conservation, Koyachi 504-1, Kashimadai, Osaki, Miyagi 989-4102, Japan; 4 Present address: Central Japan Bitterling Research Association, Hayashi 7-178-3-A503, Ogaki, Gifu 503-0015, Japan

**Keywords:** Bottleneck, Cyprinidae, conservation genetics, fish, heteroplasmy, invasive alien species, restoration

## Abstract

Genotyping of endangered species is helpful for establishing and evaluating conservation strategies. Mitochondrial sequence data was analyzed from 541 individuals of a critically endangered fish, *Acheilognathus typus* from present-day range-wide localities to re-evaluate an in-progress restoration program around Lake Izunuma-Uchinuma, Miyagi, Japan. *Acheilognathus typus* showed low sequence diversity with only eight haplotypes and π and ĥ values of 0.59129 and 0.00118 respectively. Genetic data suggests *Acheilognathus typus* is adapted to pulsed environments and prone to population flush and crash. Genotyping of populations in introduced localities revealed that their source is not from nearby localities.

## Introduction

Genotyping of endangered species offers opportunities for establishing conservation strategies, particularly for evaluating conservation unit (Crandall et al. 2000). This is especially important for freshwater fish geographically isolated by marine and land barriers. Range fragmentation by these barriers brings about geographically structured composition of populations that are potential targets of conservation programs.

**Figure 1.**
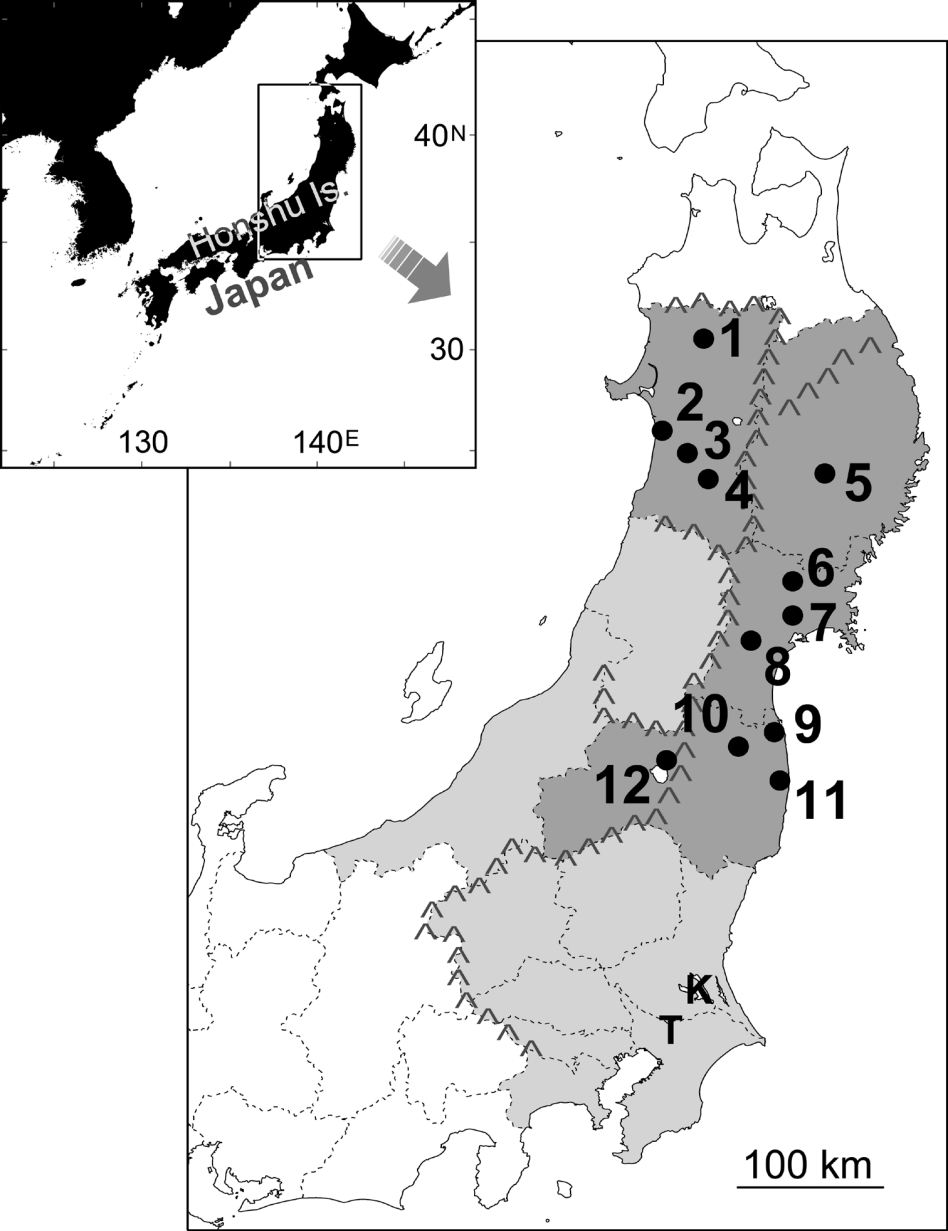
Collecting localities (**1–12**) and range contraction of *Acheilognathus typus*. Shaded areas are prefectures with past (light) and recent (dark) records. Major mountain ranges are indicated by Λ. Locality **4** contains two ponds, and **6** contains a creek and five ponds around Lake Izunuma-Uchinuma, close to each other respectively. K and T indicate locations of Lake Kasumigaura and Teganuma Lake where *Acheilognathus typus* was abundant in the past.

*Acheilognathus typus* Bleeker, 1863 is a medium-sized bitterling endemic to eastern Honshu Island, Japan. It was a common freshwater fish in shallow lakes, ponds, lowland rivers, and streams several decades ago, but desperately declined after the World War II (Nakamura 1963). Recent range contraction occurred because of frequent civil engineering works, and habitats are fragmented and unstable at present (Kitajima et al. 2004) (Fig. 1). The Ministry of Environment, Japan (2015) thus acknowledged this bitterling as a critically endangered species. In Lake Izunuma-Uchinuma, Miyagi Prefecture, Japan, this bitterling was exceptionally abundant. More than 500-individual/net/day fisheries catch was recorded in autumn 1996, but completely disappeared by 2000 (Takahashi et al. 2001) with the invasion of the largemouth bass, *Micropterus salmoides* (Lacepède, 1802). A small population near the lake was found in 2001, and restoration project of the bitterling to the lake started in 2003 including transplantation activities (Kitajima et al. 2004).

In this report mitochondrial sequence data was analyzed from 541 individuals of *Acheilognathus typus* from the present range-wide localities for re-evaluation of the restoration program. The dataset of this research is also helpful for conservation programs of this critically endangered fish.

## Materials and methods

The samples included fin-clips of 541 individuals from 18 localities collected from 2001 through 2011 (Table 1). Specimens were collected using methods including casting netting, plastic traps, or draining off pond water. Four out of 18 localities are introduced stocks (#6-5, 6-6, 7, 8). A volunteer people collaborating with the restoration project of *Acheilognathus typus* indicated that introduction took place from #6-1 into #8. Next, individuals were taken from #8 and released to #6-6, and then from #6-6 to #6-5. Introduction into #7 took place from Katsurazawa Pond in Kashimadai before the source population declined.

**Table 1.** Localities and specimens used in this study.

#	Locality	Number of individuals
1	Small pond near Kitaakita	30
2	Shiohiki Lagoon	52
3	Small pond near Nishisenboku	37
4–1	Shinzutsumi Pond in Yasumoto near Yokote	49
4–2	Pond near Yokote	20
5	Small pond in Yasawa near Hanamaki	49
6–1	Creek near Lake Izunuma-Uchinuma	39
6–2	Small pond #125 in Izunuma-Uchinuma catchment	49
6–3	Small pond #127 in Izunuma-Uchinuma catchment	18
6–4	Two neighboring small ponds #90 connected with a ditch in Izunuma-Uchinuma catchment	6
6–5	Pond #91 in Izunuma-Uchinuma catchment	48
6–6	Small pond H004 near Lake Izunuma-Uchinuma	50
7	Small pond near Kashimadai	30
8	Pond near Sendai	4
9	Creek near Soma	42
10	River near Iidate	8
11	Creek near Kodaka	8
12	Small pond near Inawashiro	2

DNA was extracted using QuickGene DNA Tissue kit on QuickGene-810 (Kurabo, Neyagawa, Japan). PCR primers were L16019 on the L-strand (GCTACCAAAGCCAGAATTCTAA) (Saitoh et al. 2004) and CrpH301D on the H-strand (GTTWARGTCCCTGATTCTATCAT) which worked for amplifying a 505 bp fragment of mitochondrial DNA encompassing 16 bp from the beginning of tRNA(Pro) gene and the left domain of control region. PCR reaction mixture of 12.5 µL contained 1 µL of template DNA, 0.96 µL of dNTP mix (2.5 nmol each), 1.2 µL of 10× Ex*Taq* buffer, 0.06 µL (0.3 U) of Ex*Taq* (Takara, Shiga, Japan), 1 µL of primers (5 pmol each), 7.28 µL of Milli-Q grade water. PCR reaction started with 3 min at 94°C followed by 35 cycles of 30 sec at 94°C, 30 sec at 55°C, 120 sec at 72°C with final extension at 72°C for 5 min. Both L and H-strand primers worked for double-stranded sequencing with BigDye terminator v.3.1 kit run on an ABI3730 sequencer (ABI, Foster City CA, USA). Sequences used in this research cover nucleotide position 15640 to 16142 of the mitochondrial genome of *Acheilognathus typus* (AB239602) (Saitoh et al. 2006). DDBJ/GenBank entries of sequences used in this research are LC148863 - LC149403.

Indices of nucleotide, haplotype, and population divergence were calculated with Arlequin v.3.5 (Excoffier et al. 2010). Parsimonious haplotype network was drawn with TCS v.1.2.1 (Clement et al. 2000). Interrelationships among populations based on pairwise net nucleotide divergence values were represented as a NJ tree with MEGA6 (Tamura et al. 2013). The net nucleotide divergence is calculated by π_xy_-(π_x_+π_y_)/2 where π_xy_ is average number of nucleotide difference between populations x and y, and π_x_ and π_y_ stand for this value between individuals within populations x and y. We inferred population expansion and contraction states calculating Tajima (1989)’s D. An overall value of D was estimated in two ways; with and without normalization of number of individuals over localities. The normalization has rationale, because absolute sample sizes obtained with different methods at different opportunities do not directly indicate differences of fish abundance among localities. A normalized sample size was set at 30 for each locality (541 / 18 localities ~ 30) and rounded up and down for minor and major haplotypes respectively to make them integer for calculation conveniences and approximation (Suppl. material 1: Table S1).

## Results

Data for *Acheilognathus typus* showed low sequence diversity. Three individuals out of 541 in total, however, indicated heteroplasmic sequence traces from both strands with doubled fluorescence peak at one or two sites each (Suppl. material 1: Fig. S1). These sites are probably of real heteroplasmy (Shigenobu et al. 2005). For analytical convenience, we phased the heteroplasmic sites into either of the two bases of non-major haplotype in the locality where the heteroplasmic individuals came from. This manipulation makes this analysis conservative by reducing haplotype skewness or by giving moderate estimation of sequence differences among haplotypes. Overall π nucleotide diversity was 0.59129 ± 0.52518, and ĥ haplotype diversity was 0.00118 ± 0.01511 upon this phasing. Upon normalization of sample size over localities, these values were 0.97484 ± 0.73356 and 0.00194 ± 0.01789 respectively.

**Figure 2.**
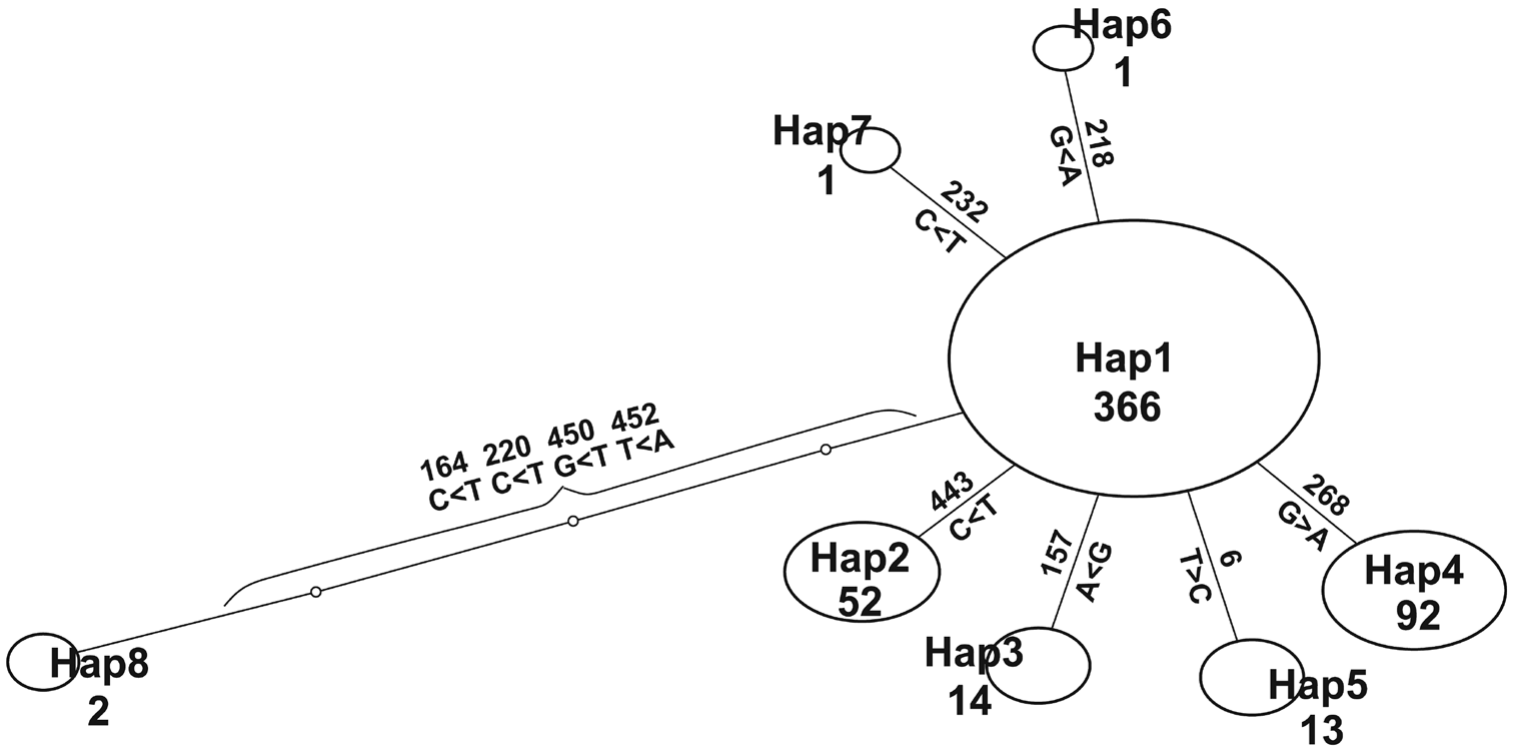
Haplotype network of *Acheilognathus typus* sequenced in this research. Small circles denote missing haplotypes. Numbers below haplotype ID stand for number of individuals observed. Numbers and bases at branches show nucleotide changes at these sites.

There were ten variable sites, and eight haplotypes appeared (Hap1-8, Table 2). A haplotype network showed a major (Hap1) and minor haplotypes connected with mostly one mismatch (Fig. 2). The major haplotype appeared in the most localities (16 of 18). Seven localities out of 18 were monotypic, and others contain at most three haplotypes. Among eight haplotypes, Hap8 is somewhat distant from others with transversions and the only haplotype detected at locality #12. This overall haplotype composition structure reflects Tajima (1989)’s D value as negative (Table 3), indicating recent population expansion under neutral evolution, though eight out of 11 polymorphic localities showed positive values.

**Table 2.** Variable sites and haplotypes.

**Nucleotide position** **Haplotype**	0 0 0 6	0 1 5 7	0 1 6 4	0 2 1 8	0 2 2 0	0 2 3 2	0 2 6 8	0 4 4 3	0 4 5 0	0 4 5 2
Hap1*	T	G	T	A	T	T	G	T	T	A
**Hap2**	T	G	T	A	T	T	G	C	T	A
**Hap3**	T	A	T	A	T	T	G	T	T	A
**Hap4**	T	G	T	A	T	T	A	T	T	A
**Hap5**	C	G	T	A	T	T	G	T	T	A
**Hap6**	T	G	T	G	T	T	G	T	T	A
**Hap7**	T	G	T	A	T	C	G	T	T	A
**Hap8**	T	G	C	A	C	T	G	T	G	T

* Identical to AB239602.

**Figure 3.**
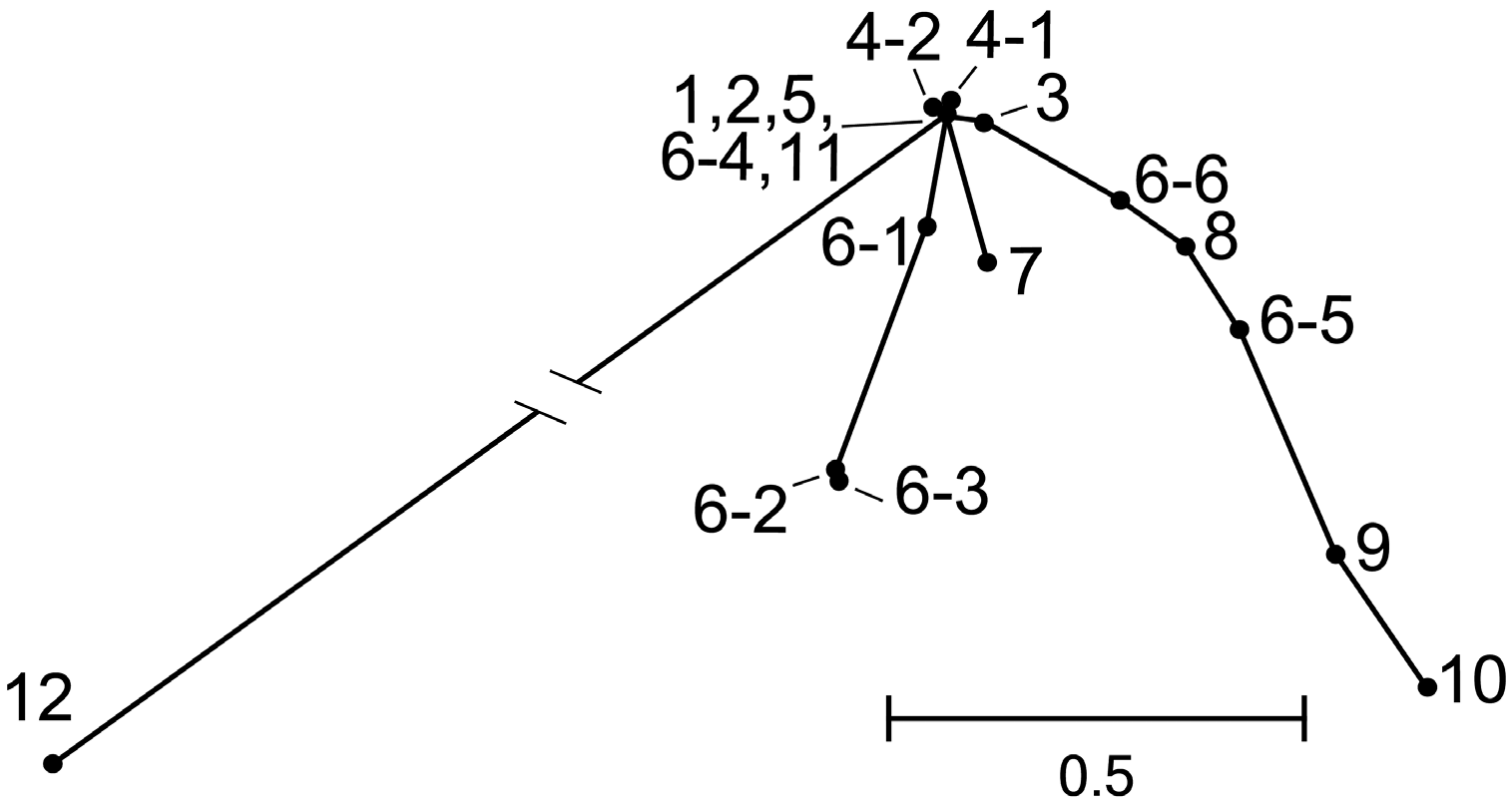
NJ tree based on net nucleotide divergence showing interrelationships among localities. Long branch between #12 with Hap8 and a node of #1, 2, 5, 6-4, 11 with Hap1 only is abbreviated and the real length is 2.4687.

Interrelationships among localities (NJ tree) based on pairwise net nucleotide divergence indicated that introduced stocks (#6-5, 6-6, 8) are close to each other, distant from others and somewhere in between localities in Fukushima (#9, 10) and others (Fig. 3). Except for these introduced stocks, those around Lake Izunuma-Uchinuma (#6-1, 6-2, 6-3, 6-4) composed of Hap2 and Hap3 with Hap1. Hap2 and Hap3 were common and characteristic to these localities. Here again, #12 was distant from others.

## Discussion

The low sequence diversity represented by both π and ĥ values with a simple haplotype network even in the fast evolving control region (Fig. 2), and negative Tajima’s D value as a whole (Table 3), indicate the population experienced a bottleneck followed by expansion in the recent past. The normalization of sample size over localities reduces skewness of haplotype composition making diversity indices higher and deviation of Tajima’s D value from zero smaller in the present case. This normalization thus makes our analysis conservative but still gave similar results. Paucity in number (1-3) of haplotypes in individual localities with positive D values is due to further subsequent bottleneck after the overall geological bottleneck and expansion (Fay and Wu 1999). The locality #12 with only a distant haplotype (Hap8) has larger population genetic distances from other localities (Fig. 3) and beyond the major mountain ranges from others (Fig. 1). We thus postulate this haplotype as a remnant in recent range contraction, though we have some reservations because of limited number of individuals examined at this locality.

**Table 3.** Haplotype composition and Tajima's D of each locality.

# \ Haplotype	Hap1	Hap2	Hap3	Hap4	Hap5	Hap6	Hap7	Hap8	D
1	30								n.a.
2	52								n.a.
3	34			3					-0.527
4-1	48					1			-1.105
4-2	19						1*		-1.164
5	49								n.a.
6-1	22	7	10						0.887
6-2	12*	33	4*						0.614
6-3	6	12							1.166
6-4	6								n.a.
6-5	23			25					1.714
6-6	29			21					1.648
7	17				13				1.578
8	2			2					1.633
9	9			33					0.681
10				8					n.a.
11	8								n.a.
12								2	n.a.
Overall	366	52	14	92	13	1	1	2	-1.246
Overall (normalized)	338	46	11	99	13	1	2	30	-0.694

* Each contains one heteroplasmic individual phased to the marked haplotype.

*Acheilognathus typus* is the sister species (Kawamura et al. 2014) and ecologically similar to *Acheilognathus longipinnis* Regan 1905 which is adapted to pulsed habitats (Odum et al. 1995) with annual flood-drought cycle (Ogawa 2011, Nishio et al. 2015).

*Acheilognathus typus* actually showed population crash killing host mussels by excess spawning in a pond (Fujimoto and Shindo 2012). We therefore postulate that *Acheilognathus typus* populations are prone to flush and crash, i.e., population bottleneck. Overall haplotype composition and negative Tajima’s D yet positive in individual localities (Table 3) support our hypothesis.

Fish species that experience frequent population flush and crash events need wider habitats that allow spatially various phases in environmental fluctuation. In such habitats, those fish species like *Acheilognathus typus* will sustain as metapopulations in which constituent subpopulations temporarily work as source or sink, and vice versa. Lake Izunuma-Uchinuma (5 km^2^) of 20 years ago, Lake Kasumigaura (172 km^2^) of more than 30 years ago, and Teganuma Lake (4 km^2^) of 45 years ago were such good habitats for *Acheilognathus typus* (Fig. 1). Present-days known and introduced habitats are, however, small ponds (< 1 ha) in many cases, and population sustainability is questionable.

In Lake Izunuma-Uchinuma, invasion and establishment of largemouth bass have inhibited recovery of *Acheilognathus typus* population (Takahashi et al. 2001). The largemouth bass exterminates bitterlings by heavy predation shortly (Fujimoto et al. 2009). The largemouth bass cleanup efforts with intensive catch at all life stages by people nearby, however, reduced the bass stock in the lake drastically, and cyprinid fishes are recovering (Ueda 2013). What were and what should be the genetic characteristics of the past and recovering *Acheilognathus typus* population in the lake? Remnant populations can be characterized with specific common haplotypes (Hap2 and Hap3) (Table 3). We postulate four localities and others uncovered, if any, represent as a whole the past genetic composition of the lake population (branch of #6-1, 6-2, 6-3 in Fig. 3), and recovery of this population is the goal of the restoration program.

Under this circumstance, a good practice for restoring *Acheilognathus typus* population may be to propagate them at first in ponds where the bass is absent in the lake catchment. Propagated *Acheilognathus typus* then hopefully flows out from the ponds to the lake. The anticipated outflow would be a natural experiment whether *Acheilognathus typus* population could establish in the lake where a small stock of the bass still remains.

Re-established stock at locality #6-5 in the lake catchment, however, may be inconvenient for recovery of the past *Acheilognathus typus* population in the lake. Haplotype composition of that stock is similar to those in Fukushima (#9, 10) (Fig. 3, Table 3). The source of that stock is unknown and not from those nearby Lake Izunuma-Uchinuma. Outflow from that pond, if any, may change genetic composition of expected recovered population in the lake.

Introduction of *Acheilognathus typus* into the ponds #6-6 from #8 and then from #6-6 to #6-5 took place when population at #6-1 declined temporarily. At that time it was not possible to introduce *Acheilognathus typus* from #6-1 which was the only known locality near Lake Isunuma-Uchinuma. Pond #8 was then selected for the source of pond #6-6 based on the unverified information that it was introduced from #6-1, but it was misleading.

A lesson from above is importance of intensive survey of habitats, both known and unknown, before introduction. Population at #6-1 recovered in 2011 (Table 1) indicating actual population crash and flush. Populations in a few ponds in the lake catchment were also found after intensive survey (#6-2, 6-3, 6-4). We conducted this genetic research after the finding of these populations, and identified the introduced populations near the lake (#6-5, 6-6) were not representatives of the past Lake Izunuma-Uchinuma population.

## Supplementary materials

Supplementary material 1 Table S1, Figure S1 Data type: sample data Explanation note: **Table S1.** Normalized haplotype composition of localities.
**Figure S1.** Sequence traces of both directions with double-peaked sites. This dataset is made available under the Open Database License (http://opendatacommons.org/licenses/odbl/1.0/). The Open Database License (ODbL) is a license agreement intended to allow users to freely share, modify, and use this dataset while maintaining this same freedom for others, provided that the original source and author(s) are credited.Kenji Saitoh, Kentaro Shindo, Yasufumi Fujimoto, Kiyotaka Takahashi, Tetsuo Shimada
