# Supplementary material for: Mitochondrial genotyping of an endangered bitterling Acheilognathus typus (Cyprinidae)
Source: Zookeys. 2016 Oct 11;(623):131–41. doi: 10.3897/zookeys.623.8981 (PMC5096401; doi:10.3897/zookeys.623.8981)
Supplement: Supplementary material 1 — Table S1, Figure S1 [file zookeys-623-131-s001.pdf]

Mitochondrial genotyping of an endangered bitterling *Acheilognathus typus*

Kenji Saitoh, Kentaro Shindo, Yasufumi Fujimoto, Kiyotaka Takahashi, Tetsuo Shimada

Supplementary Data

Table S1. Normalized haplotype composition of localities.

# \ Haplotype	Hap1	Hap2	Hap3	Hap4	Hap5	Hap6	Hap7	Hap8
1	30							
2	30							
3	27			3				
4-1	29					1		
4-2	28						2	
5	30							
6-1	16	6	8					
6-2	7	20	3					
6-3	10	20						
6-4	30							
6-5	15			15				
6-6	17			13				
7	17				13			
8	15			15				
9	7			23				
10				30				
11	30							
12								30
overall	338	46	11	99	13	1	2	30

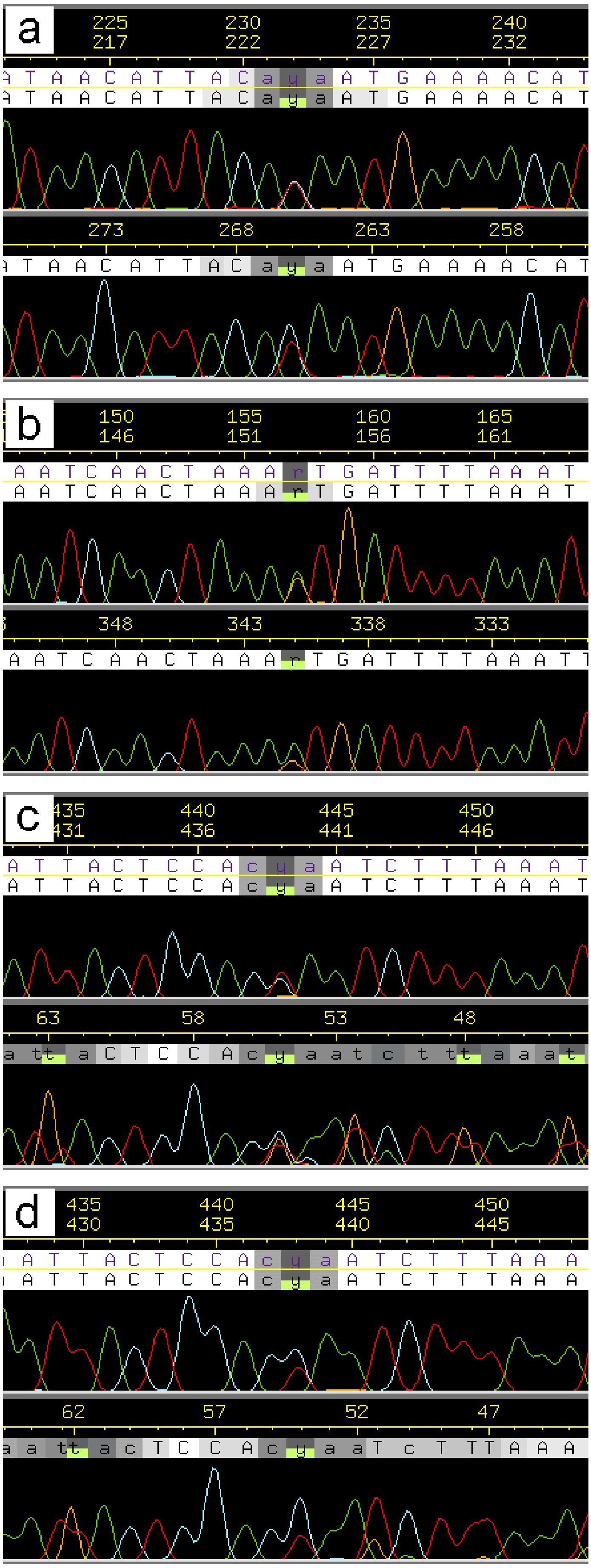

Figure S1. Sequence traces of both directions with double-peaked sites. An individual collected at locality #4-2 (a) showed C/T at 232th site, and this site was phased to C. A/G at 157th (b) and C/T (c) at 443th sites of an individual from locality #6-2 were phased to A and T respectively. C/T at 443th site of another individual from locality #6-2 was phased to T. C blobs near the start site of reverse reads (exhibited as G blobs on reverse complement traces on lower panels of c and d) were ignored.
